# Competing or Interactive Effect Between Perceived Response Efficacy of Governmental Social Distancing Behaviors and Personal Freedom on Social Distancing Behaviors in the Chinese Adult General Population in Hong Kong

**DOI:** 10.34172/ijhpm.2020.195

**Published:** 2020-10-18

**Authors:** Yanqiu Yu, Joseph Tak Fai Lau, Mason Man Chun Lau

**Affiliations:** Centre for Health Behaviours Research, Jockey Club School of Public Health and Primary Care, The Chinese University of Hong Kong, Hong Kong SAR, China.

**Keywords:** COVID-19, Social Distancing, Response Efficacy, Freedom, China, Interaction

## Abstract

**Background:** Uptake of social distancing behaviors may be determined by a combination of individual perceptions and social values. The study investigated (1) the associations between individual perception of perceived response efficacy and social distancing behaviors, (2) the association between social value of perceived freedom infringement and social distancing behaviors, and (3) whether perceived freedom infringement would moderate the association between perceived response efficacy and social distancing behaviors.

**Methods:** A cross-sectional telephone survey interviewed 300 adults in the Hong Kong adult general population during April 21-28, 2020. The instruments of social distancing behaviors, perceived response efficacy, and perceived freedom infringement assessed the frequencies of practicing seven types of social distancing behaviors in the past week, perceived response efficacy of four types of governmental social distancing measures/instructions, and a 5-point Likert scale item on perceived infringement on personal freedom regarding a governmental social distancing measure of banning gatherings of >4 people in public areas. Linear regression adjusted for background factors was performed; the interaction term of perceived response efficacy × perceived freedom infringement was tested.

**Results: **About 40.4%-83.0% of the respondents practiced various types of social distancing behaviors; 57.3%-75.0% perceived response efficacies of related governmental measures; about 20% showed perceived freedom infringement. Perceived response efficacy, but not perceived freedom infringement, was independently and positively associated with social distancing behaviors. Perceived freedom infringement significantly moderated the association between perceived response efficacy and social distancing behaviors; such a positive association was significant at higher (those scored "extremely agree"), but not lower (those scored "extremely disagree"), levels of perceived freedom infringement.

**Conclusion:** Perceived response efficacy is a potential determinant of social distancing. However, the strength of such an association may be modified by opposing social values about personal freedom. Future studies are warranted to verify above findings and explore other potential determinants.

## Background

**Key Messages**

Implications for policy makers

- The levels of uptake of social distancing behaviors and perceived response efficacy of governmental social distancing measures were high in Hong Kong.
- Improvement on perceived efficacy of related governmental measures may increase the uptake of social distancing behaviors directly.
- Around one fifth of participants considered the restriction of gathering size in public areas an infringement of personal freedom.
- The effect of perceived infringement of freedom did not compete against perceived response efficacy onto the uptake of social distancing behaviors.
- The positive effect of perceived response efficacy on the uptake of social distancing behaviors was significant only among those with a stronger, but not weaker, perception about freedom infringement.

Implications for the public

Social distancing is a controversial public health measure that involves both public health benefits (eg, reduction of transmission of coronavirus disease 2019 [COVID-19]) and conflicting values (eg, perceived infringement of personal freedom). In Hong Kong SAR, China, the levels of perceived response efficacies were relatively high, which might contribute to the relatively high levels of the uptake of various social distancing behaviors. In contrast, about one fifth of participants had concerns about the infringement of personal freedom due to a social distancing measure, but such personal value had no competing effect against perceived response efficacy onto social distancing behaviors. Notably, perceived infringement of personal freedom moderated the association between perceived response efficacy and social distancing behaviors, indicating that perceived response efficacy only affected people who had stronger, but not weaker, perceptions about freedom infringement. The findings suggest that structural factors may modify the effect of cognitive factors on determining social distancing behaviors.

Social distancing has become one of the key global measures used to control the coronavirus disease 2019 (COVID-19) pandemic.^1-6^ The World Health Organization (WHO) suggests using the term “physical distancing” instead of “social distancing,” as it is the physical distance that prevents transmission while people can remain socially connected via the Internet or other means. Social distancing had been used to reduce transmission of severe acute respiratory syndrome (SARS), swine flu (H1N1), and Ebola virus disease.^7,8^ In face of the high infectivity and asymptomatic transmission features of COVID-19,^9,10^ social distancing has particular significance. Social distancing during the COVID-19 pandemic includes multiple legal measures such as suspending classes/religious gatherings/sports events, banning people going out without important reasons, closing bars and restaurants, allowing people to work from home, and restricting size of gatherings (eg, four or eight) within distances of one to two meters.^1-6^ Together with the prevalent use of personal preventive measures (face-mask and good hand hygiene), social distancing should be able to curb the spread of the virus.

 The effectiveness of the governmental social distancing measures, however, depends on the level of compliance in the general population, which cannot be taken for granted (eg, only around 40% in the United States according to a pre-print^11^). Keeping social distancing as part of a normal lifestyle is equally essential, as governmental social distancing measures cannot control all interpersonal contacts in public and private arenas. For instance, in many places, people are not required to stay at home (eg, many cities in China and the United States) and people are free to join large social gatherings in private settings. Thus, like other personal preventions, social distancing is very often a volitional personal choice that is subjected to multiple cognitive determinants. There is a dearth of published studies that investigated such determinants, although a few preprints reported factors such as income, empathy, norms, and boredom.^12-15^

 Perceived response efficacy is a potential key factor of social distancing behaviors. It refers to a person’s belief about whether the recommended action will actually avoid the threat.^16^ In the present study, it refers to the perceived effectiveness of the governmental social distancing policies in controlling the COVID-19 pandemic. Various behavioral health theories have highlighted the significance of perceived response efficacy in determining health-related behaviors. For instance, perceived response efficacy is often seen as a part of the construct of perceived benefits in the Health Belief Model,^17^ which states that a person would perform a health-related behavior if he/she perceives high susceptibility/severity related to the health problem and high levels of perceived benefit/self-efficacy (confidence)/cue to action and a low level of barrier. The Protection Motivation Model of fear appeal also involved perceived response efficacy, which represents coping appraisal process regarding a threat; jointly with self-efficacy, it determines whether an individual would take up a health-related behavior.^16^ Empirically, ample studies have shown that perceived response efficacy of preventive measures was significantly associated with uptake of such behaviors during H1N1 and H5N1 outbreak periods.^18-21^ The very limited literature suggested that the same was probably true regarding social distancing during the COVID-19 pandemic. A Korean study showed that perceived response efficacy was positively associated with social distancing behaviors (reducing the use of public transportation, keeping away from crowded places, and postponing/canceling social events)^22^; another Iranian study reported that perceived response efficacy was positively associated with protection motivation to conduct COVID-19-related preventive behaviors among health workers, which was associated with the actual behaviors.^23^ A study showed that perceived efficacy of governmental actions related to COVID-19 varied across countries (Norway >Israel >the United States >Colombia >Brazil >Germany), and it was associated with the number of individuals’ preventive actions.^24^ Our literature search cannot find other studies that investigated the relationships between perceived response efficacy of governmental social distancing measures and individual social distancing behaviors related to COVID-19. Such studies are important as perceived response efficacy can be increased by health promotion.

 According to the socio-ecological model, structural factors are also strong determinants of health-related behaviors.^25-27^ There were severe disputes about the implementation of social distancing measures within some governments and countries, such as the United States.^6,28^ This disputes were partly due to the obviously severe negative impacts of social distancing on the recessing economies in many countries.^29^ As a structural factor, inconsistency between social values and a public health control measure may reduce public engagement in the behavior (social distancing in this case). For instance, some studies conducted in Africa reported that trust toward the government was associated with preventive behaviors against Ebola.^30-32^ A few studies looked at the role of values and various community responses during the COVID-19 pandemic. One of them showed that collectivism was associated with a stronger risk perception of COVID-19 and weaker psychological maladjustment.^33^ Freedom is highly valued. A preprint reported that social distancing policies had reduced overall movement and travel at both individual and state levels in the United States.^34^ Social distancing, including the commonly exercised mandatory measure of gathering size restriction, may be perceived as an infringement of personal freedom in Hong Kong. It changed from a restriction of >4 people (from March 29 to May 4, 2020 when the study was conducted) to >8 people (from May 5 to June 18, 2020) according to the severity of the outbreak, and it has a strong impact on Hong Kong people’s daily life, as it is a very dynamic and densely populated city that people have to meet frequently for social and work reasons. The belief that social distancing infringes on individual freedom may compromise compliance with social distancing measures. In some countries (eg, the United States, Britain, and Italy), protests were held against the implementation of governmental social measures, stating that it limits personal freedom.^35,36^ In a previous study, the view that “the government interferes far too much in our everyday lives” was negatively associated with perceived risk for COVID-19.^37^ Our literature review, however, cannot identify any study that investigated the relationship between the value of personal freedom and social distancing behaviors.

 The present study investigated the associations between a personal-level facilitating factor (perceived response efficacy of social distancing measures) and a structural prohibiting factor (the perception that the mandatory restriction of gathering size in public areas infringes personal freedom) and the levels of social distancing behaviors in the past week. Besides looking at whether the two constructs would exhibit independent competing associations with social distancing behaviors, statistical significance of the moderation effect of perceived infringement of personal freedom for the association between perceived response efficacy and levels of social distancing behaviors was also tested in this study. Three hypotheses were tested: (1) perceived response efficacy would be positively associated with social distancing behaviors; (2) perceived infringement of personal freedom would be negatively associated with social distancing behaviors; (3) a significant statistical interaction would exist (ie, the association between perceived response efficacy and social distancing behaviors would be stronger among those with higher levels than those with lower levels of perceived infringement of personal freedom).

## Methods

###  Study Design 

 A random telephone survey was conducted among Hong Kong Chinese adults (aged ≥18 years) during April 21-28, 2020; 300 participants were anonymously interviewed between 6-10:30 pm (10 to 15 minutes) by experienced interviewers to avoid over-sampling non-working individuals. Telephone numbers were randomly drawn from the most updated residential telephone directory. The household member whose birthday was closest to the interview date was invited to join the study if the household had more than one eligible prospective participants. The participants were briefed by the interviewer about the background of the study and the option to skip questions or quit any time without being questioned. The questions were asked in the fixed sequence of perceptions (perceived response efficacy and perceived infringement of personal freedom) followed by behaviors (social distancing). Unanswered telephone calls were given at least three attempts before being classified as invalid. Unavailable eligible participants were contacted again; appointments were made if necessary. Verbal informed consent was obtained prior to commencement of the interviewers; the interviewer signed on a form to plead that they had explained the study fully to the participants and answered their questions. Of the eligible participants, 54.3% (n = 300) completed the interviews. No incentives were given to the participants.

###  Measures

####  Background Variables 

 Information about socio-demographics and the perceived need to have close physical contacts during work (1 = extremely low/not applicable to 5 = extremely high) was collected.

####  Social Distancing Behavior Scale

 The 7-item scale assessed the levels of social distancing behaviors in the past week, by asking about self-reported frequencies of avoiding: (*i*) going out unless necessary, (*ii*) social gatherings, (*iii*) meeting with acquaintances, (*iv*) visiting crowded places, (*v*) being within 1.5 meters with other people, (*vi*) gatherings involving >4 persons, and (*vii*) using public transportation. The items were measured with 5-point Likert scales (1 = never to 5 = always); higher scores indicated higher levels of social distancing behaviors. Confirmatory factor analysis was conducted to test the one-factor model of the Social Distancing Behavior Scale, which found satisfactory goodness-of-fit indices after taking into account of covariance between item error terms (Chi-square/*df*= 1.28, *P* <.001, comparative fit index [CFI] = 0.99, normed fit index [NFI] = 0.98, and root mean square error of approximation [RMSEA] = 0.07). The Cronbach’s alpha was 0.82.

####  Perceived Response Efficacy Scale of Governmental Social Distancing Measures 

 The 4-item scale assessed the level of perceived response efficacy for various governmental social distancing measures/instructions, including (*i*) banning gatherings of >4 persons in public areas, (*ii*) closure of entertainment venues (eg, bars, cinemas, beauty salons, night clubs, and fitness centers), (*iii*) closure of public facilities (eg, library and sports field), and (*iv*) banning people going out unless necessary. The items were rated with 5-point Likert scales (1 = extremely low to 5 = extremely high); higher scores indicated higher levels of perceived response efficacy. The confirmatory factor analysis on the one-factor model of the perceived response efficacy scale (PRES) showed satisfactory goodness-of-fit [Chi-square/*df*= 3.54, *P* <.001, CFI = 0.99, NFI = 0.99, and RMSEA = 0.09]. The Cronbach’s alpha was 0.82.

####  Perceived Infringement of Personal Freedom Due to Social Distancing

 One item assessed how much the participants agreed with the statement “The banning of gatherings of >4 persons in public areas by the government infringes on my right for personal freedom” (1 = extremely disagree to 5 = extremely agree).

###  Statistical Analysis

 Simple and multivariable (adjusted for background factors) ordinary least square linear regression analysis were conducted to test the associations among background factors, PRES, perceived infringement of freedom, and the level of social distancing behaviors. Standardized regression coefficients (*β*) were presented in this report. It represents the change in the number of standard deviation (SD) in the dependent variable, given one unit change in the independent variable of concern. The moderation effects of perceived infringement of freedom on the association between PRES and social distancing behaviors were examined by testing the significance of the interaction term of PRES × perceived infringement of freedom, after adjustment of background factors. To illustrate the meaning of the moderation effect graphically, the modeled regression lines between perceived response efficacy and social distancing behaviors were plotted for two scores of the perceived freedom infringement scale with endorsement of “extremely disagree (value = 1)” and “extremely agree (value = 5).” The moderation effect and the graphical presentation were generated by Process Macro^38^ in SPSS 21.0. The statistically significant level was defined as two-sided *P *<.050. The term of marginal significance was used when the results were close to but did not meet the traditional convention for statistical significance (*P *<.050), ie, when.050 < *P *<.100.

## Results

###  Descriptive Statistics

####  Background Characteristics of the Participants

 Among the 300 participants, close to or more than half were females (67.3%), aged >55 years old (47.4%), and being married/cohabitating with someone (65.3%). About 1/4 received tertiary education or above (25.6%) and perceived moderate to extremely high need to have close physical contacts (<1.5 meters) during work (26.7%) (Table 1).

**Table 1:** Descriptive statistics of participants (n = 300)

	**No.**	**%**
**Background Variables**		
Gender		
Male	98	32.7
Female	202	67.3
Age		
18-35	53	17.7
36-55	102	34.0
56-65	65	21.7
> 65	77	25.7
Missing data	3	1.0
Marital status		
Single/separated/divorced/widow/widower	104	34.7
Cohabitation/married	196	65.3
Educational level		
≤Primary school	53	17.7
Middle school/matriculation	169	56.3
≥College	77	25.6
Missing data	1	0.3
Perceived need to have close physical contacts during work		
Not applicable/extremely low/low	220	73.3
Moderate/high/extremely high	80	26.7
**Perceived Response Efficacy of Governmental Social Distancing Measures**		
Prohibition of gatherings of >4 persons		
Extremely low	9	3.0
Low	19	6.3
Moderate	80	26.7
High	123	41.0
Extremely high	69	23.0
Closure of entertainment venues		
Extremely low	7	2.3
Low	14	4.7
Moderate	54	18.0
High	127	42.3
Extremely high	98	32.7
Closure of public facilities		
Extremely low	11	3.7
Low	34	11.3
Moderate	58	19.3
High	108	36.0
Extremely high	89	29.7
Prohibition of people going out unless necessary		
Extremely low	29	9.7
Low	32	10.7
Moderate	67	22.3
High	73	24.3
Extremely high	99	33.0
Perceived Infringement of Personal Freedom		
Extremely disagree	78	26.0
Disagree	107	35.7
Neutral	58	19.3
Agree	33	11.0
Extremely agree	24	8.0

####  Social Distancing Behavior Scale

**Figure 1:**
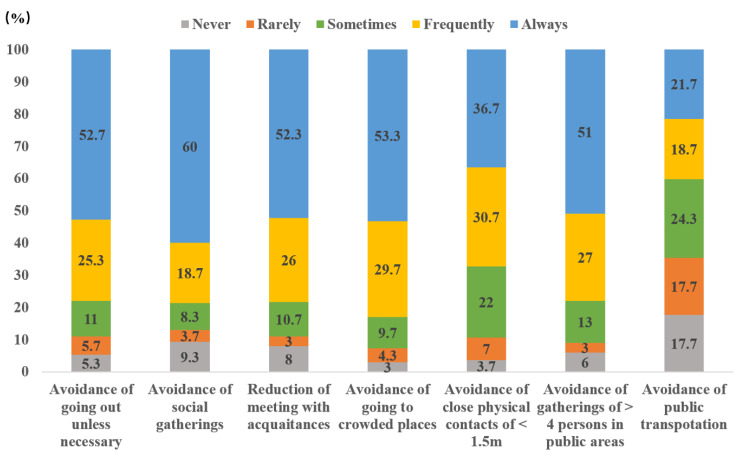
Distributions of the 7 Types of Social Distancing Behaviors.

 The mean (SD; range) score of the Social Distancing Behavior Scale was 27.8 (5.7; 7-35). Descriptive statistics of compliance with specific social distancing behaviors are presented in Figure 1. Close to or more than 80% of the participants frequently or always avoided (*i*) going out unless necessary (78.0%), (*ii*) social gatherings (78.7%), (*iii*) meeting with acquaintances (78.3%), (*iv*) visiting crowded places (83.0%), (*v*) being within <1.5 meters with other people (67.4%), and (*vi*) gatherings involving >4 persons (78.0%). Fewer people, however, frequently/always avoided using public transportation (40.4%).

####  Perceived Response Efficacy of Governmental Social Distancing Measures

 The mean (SD; range) score of the PRES was 15.1 (3.5; 4-20). Over half of the participants considered specific governmental measures to be highly or extremely highly efficacious, including (*i*) banning gatherings of >4 persons (64.0%), (*ii*) closure of entertainment venues (75.0%), (*iii*) closure of public facilities (65.7%), and (*iv*) banning people going out unless necessary (57.3%) (Table 1).

####  Perceived Infringement of Personal Freedom

 Nearly one-fifth of the participants agreed or extremely agreed with the statement that banning gatherings involving >4 persons infringed on his/her right for personal freedom (19.0%) (Table 1).

###  Simple Regression Analysis for the Associations Between Background Factors and Perceived Response Efficacy/Perceived Infringement of Personal Freedom

 The results are shown in Table 2. Age and current marital/cohabitation status were positively associated with perceived response efficacy, while those with the secondary educational level were less likely than others to perceive response efficacy. Those with older age and perceived higher need to have close physical contacts during work were less likely than others to perceive infringement of personal freedom. The other associations were statistically non-significant. The background factors listed in Table 2 altogether explained 22.1% and 5.9% of the variance (*R*^2^) of perceived response efficacy and perceived infringement of personal freedom, respectively (statistics not shown in the tables).

**Table 2:** Simple Linear Regression Analysis Among the Studied Variables (n = 300)

	**Perceived Response Efficacy **		**Perceived Infringement of Personal Freedom**		**Social Distancing Behaviors**	
	***β***	***P***	***β***	***P***	***β***	***P***
**Background variables**						
Gender						
Male	Ref		Ref		Ref	
Female	-0.06	.342	-0.01	.963	0.07	.202
Age						
18-35	Ref		Ref		Ref	
36-55	0.36	<.001	-0.14	.075	0.11	.135
56-65	0.34	<.001	-0.14	.059	0.35	<.001
>65	0.57	<.001	-0.23	.003	0.21	.004
Marital status						
Single/separated/divorced/widow/widower	Ref		Ref		Ref	
Cohabitation/married	0.14	.014	-0.09	.130	0.12	.045
Educational level						
≤Primary school	Ref		Ref		Ref	
Middle school/matriculation	-0.25	.001	0.03	.693	-0.10	.203
≥College	-0.12	.124	0.03	.748	-0.08	.337
Perceived need to have close physical contacts during work						
Not applicable/extremely low/low	Ref		Ref		Ref	
Moderate/high/extremely high	0.04	.505	-0.12	.046	-0.09	.133
**Perceived response efficacy **	-	-	-	-	0.19	.001
**Perceived infringement of personal freedom**	-	-	-	-	-0.10	.097

###  Correlation Between the 2 Independent Variables 

 Perceived response efficacy was negatively correlated with perceived infringement of personal freedom (*r *= -0.34, *P* <.001)

###  Factors of Social Distancing Behaviors

 The simple regression (one independent variable and one dependent variable in each model) results showed that two of the background factors [older age (>55 years versus 18-35 years) and current marital/cohabitation status] and perceived response efficacy were associated with higher levels of social distancing behaviors (*β* = 0.19; *P *=.001), while perceived infringement of personal freedom were inversely and marginally (.050 < *P* <.100) associated with social distancing behaviors (*β* = -0.10; *P *=.097) (see Table 2).

 The results of the multivariable analysis that adjusted for all the background factors are presented in Table 3. First, perceived response efficacy was significantly and positively associated with social distancing behaviors (*R*^2^ = 0.115; *β* = 0.15; *P *=.018; Model 1). Second, the association between perceived infringement of personal freedom and social distancing behaviors was statistically non-significant (*R*^2^= 0.103; *β* = -0.07; *P *=.200; Model 2). Third, when both perceived response efficacy and perceived infringement of personal freedom were present in the same model (Model 3), the adjusted analysis yielded a R-square value of 0.116. In that model, perceived response efficacy (*β* = 0.14; *P *=.038) but not perceived infringement of personal freedom (*β* = -0.63; *P *=.528) was significantly associated with social distancing behaviors.

**Table 3:** Multivariable Linear Regression Analysis for Testing Factors of Social Distancing Behaviors and the Interaction Between the 2 Factors (n = 300)

	**Social Distancing Behaviors**			
	**Model 1**	**Model 2**	**Model 3**	**Model 4**
	***β***	***β***	***β***	***β***
Perceived response efficacy	0.15 (*P* =.018)		0.14 (*P* =. 038)	-0.09 (*P* =.463)
Perceived infringement of freedom		-0.07 (*P* =.200)	-0.63 (*P* =.528)	-0.51 (*P* =.026)
Perceived response efficacy × Perceived infringement of freedom				0.46 (*P* =.032)
*df1, df2*	(9, 290)	(9, 290)	(10, 289)	(11, 288)
*F*	4.196 (*P* <.001)	3.706 (*P* <.001)	3.809 (*P* <.001)	3.927 (*P* <.001)
*R* ^ 2 ^	0.115	0.103	0.116	0.130

###  Testing Moderation Effect of Perceived Infringement of Personal Freedom on the Association Between Perceived Response Efficacy and Social Distancing Behaviors

**Figure 2:**
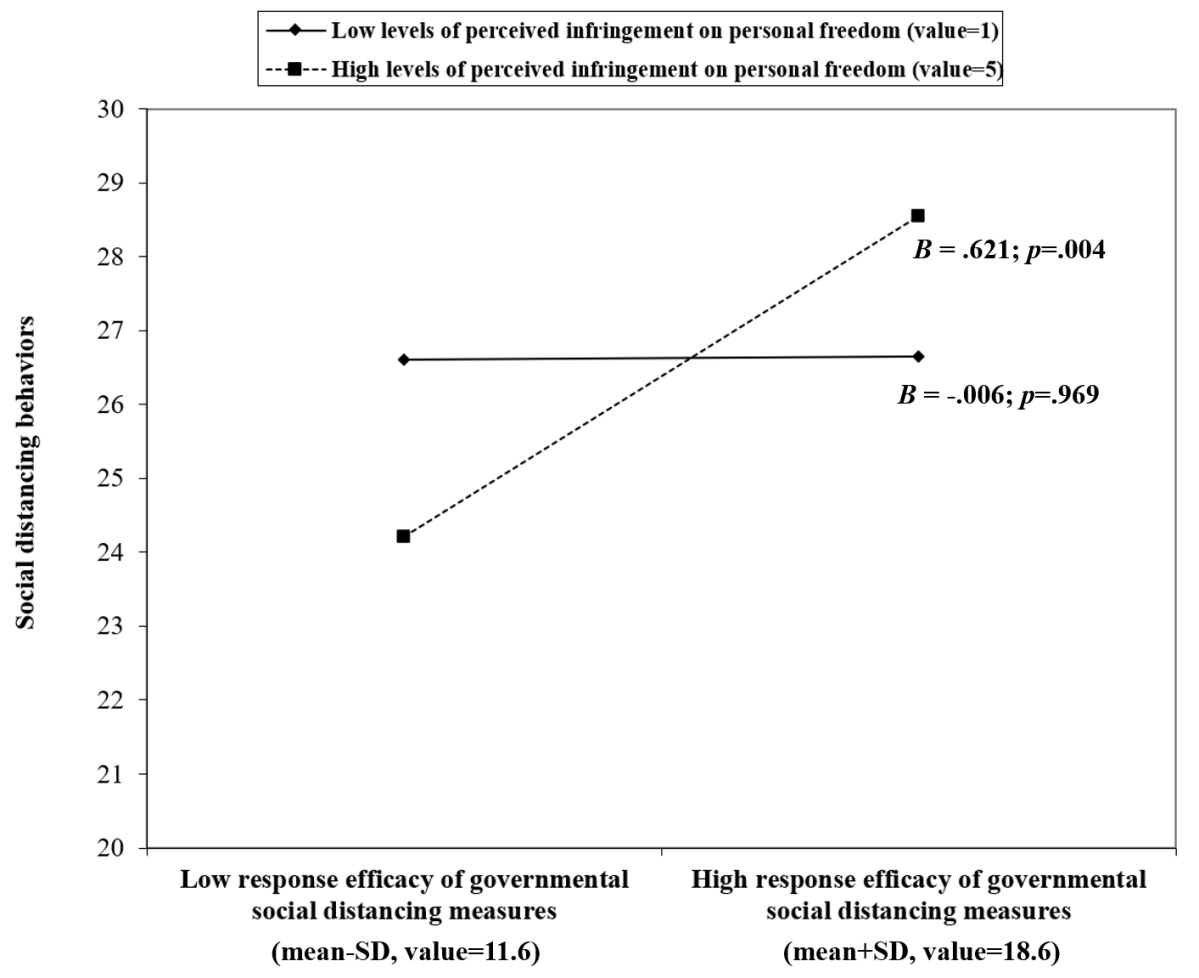
Graphical Representation of the Moderation Effect. Abbreviation: SD, standard deviation.

 In Model 4 of Table 3, the main effect of perceived infringement of personal freedom (the moderator) and the interaction term (perceived infringement × perceived response efficacy), but not the main effect of perceived response efficacy, were statistically significant. The graphical representation of the interaction effect is shown in Figure 2. The strength of the association between perceived response efficacy increased with the level of perceived infringement of personal freedom. Specifically, among those who “extremely agreed” with the statement about perceived infringement of freedom, perceived response efficacy was positively associated with social distancing behaviors (*B *= 0.621; *P *=.004); the association was non-significant among those who “extremely disagree” with the statement about perceived freedom infringement (*B *= 0.006; *P *=.969). It is seen from Figure 2 that the slope of those with perceived infringement of personal freedom was substantial. It implies that an increase in 2.35 points in social distancing scale score (with the scale range 28 points of the Social Distancing Behavior Scale) would be resulted when the PRES score increased by 3.5 points, which is equivalent to one SD (within the range of 16 points of PRES). In comparison, the slope of those who did not perceive freedom infringement, as seen from Figure 2, was almost flat.

## Discussion

 Social distancing has been used as a major global strategy to control the COVID-19 pandemic. The scale and scope of the related measures were unprecedented, ranging from lockdowns to restriction of gathering size in public areas, and involved fines and even imprisonment. The majority of the participants (about 3/4 or more) had practiced various means of social distancing (eg, avoiding social gatherings). However, less seemed to be able to practice physical distancing (eg, keeping a physical distance of >1.5 meters and avoiding using public transportation). This is understandable in the context of Hong Kong where there was no lockdown and working and daily activities were in general normal. The densely populated environment and reliance on public transportation limited the extent of physical distancing. Thus, the level of physical distancing in a city may depend on contextual factors such as population density and utilization rate of public transportation. It is important to compare the findings with other countries that vary in population density and other epidemic and policy features of COVID-19 (eg, the number of cases and the degree of lockdown). Policy-making on social distancing measures need to take the physical and social contexts into account.

 In Hong Kong, the relatively low number of COVID-19 infection cases (1103 cases as of June 5, 2020) could be attributed to the almost universal facemask use,^39,40^ extensive screening, contact tracing, and quarantine policies. Voluntary and mandatory social distancing should have also played an important role as all these measures reinforce each other. Although social distancing was common, about 1/4 or more of the participants did not observe such measures. Thus, health promotion is still necessary. Younger and single participants were less likely than their counterparts to practice social distancing as they may have more active social and work life. Sex and education were not significantly associated with social distancing. The reasons require further study, as such factors might be both positively and/or negatively associated with multiple factors of social distancing.

 More than half of the participants perceived relatively high response efficacy regarding various public health measures. About 3/5 to 3/4 of the participants endorsed response efficacy of restricting gathering size of ≤4, closure of entertainment venues and public facilities (eg, playgrounds and museums), and avoidance of going out unless necessary. It is imperative that good compliance of such policies requires good perceived response efficacy in the general population. In the present study, it is noteworthy that perceived response efficacy of the social distancing measures was significantly associated with the level of social distancing. The findings corroborated previous studies that response efficacy of public health measures may increase levels of uptake of personal preventive behaviors.^18-21^ The study adds similar evidence to the literature of COVID-19 studies. It also partially supports related behavioral health theories postulating perceived response efficacy as a determinant of preventive behaviors (eg, the Health Belief model). International comparisons are important as the levels of perceived response efficacy seem to vary tremendously across countries and among political leaders, who faced tough decisions of balancing between social distancing and economic recovery.

 Given the positive association between perceived response efficacy and social distancing behaviors, future studies should explore whether fostering perceived response efficacy via health promotion would increase social distancing during the COVID-19 pandemic. If the findings were affirmative, improvement could be made as the endorsement rate of perceived response efficacy was only about 2/5 in this study. Such health promotion may need to pay attention to the younger people and those with secondary education level as they tended to report lower perceived response efficacy. Younger people might perceive low response efficacy due to their lower perceived risk and perceived susceptibility regarding COVID-19.^41^ Besides, younger people in Hong Kong showed very low levels of trust toward the government due to the ongoing social movement (unpublished data). The lack of trust might have weakened young people’s perceived response efficacy.

 Furthermore, we need to know more about the actual response efficacy of social distancing measures, as, except for a few observational studies conducted during H1N1 and H5N1 outbreaks,^18-21^ solid evidence about the efficacy of social distancing in controlling outbreaks of emerging infectious diseases seems unavailable. It is noteworthy that the setting up and/or relaxation of social distancing rules (eg, one-to-two-meter physical distancing and gathering size of ≤4/≤8) might actually be arbitrary and certainly not evidence-based. Although some related modeling data looked at the effect of social distancing on the spread of COVID-19,^11,42^ contextual factors (eg, compliance, screening policies, and the number of cases) might affect the models’ accuracies. It is also warranted to understand the effectiveness of various means of social distancing in populations that have various facemask usage rates.

 Health promotors need to take structural factors that facilitate or compromise social distancing behaviors into consideration. It is noteworthy that about 1/5 of the participants believed that the local policy of banning gatherings of size >4 in public areas had infringed their freedom. Understandably, perceived freedom infringement was found to be negatively associated with perceived response efficacy. First, according to cognitive emotion regulation theory, those who perceived infringement of freedom may have negative attitudes about social distancing; the negative emotion may foster negative cognitions about the social distancing measures.^43^ Second, age may be a potential confounder as younger people tended to perceive both lower response efficacy and stronger infringement of personal freedom. Third, the concern about personal freedom might be even stronger in Western societies, which has a tradition of emphasizing the protection of personal freedom.^44^ It is interesting to point out that Hong Kong has cultural heritages from both the East and the West. A majority (90.6%) of the Hong Kong residents are Chinese; most of their parents or grandparents immigrated from mainland China during the 1950s and 1960s. Traditional Chinese culture is thus vital. In parallel, the city was a British colony prior to 1997. As a result, the education, legal, and medical systems were (are) under strong British influences. Hong Kong has also evolved herself into one of the most internationalized cities. Thus, both the traditional Chinese collectivism and the western belief of personal freedom have deep roots in the general population. It is possibly that Hong Kong has a ‘middle ground’ position in the spectrum of individualism versus collectivism. Overall, we thus believe that perception of freedom infringement related to social distancing and its impact on social distancing in the Hong Kong general population might be stronger than those of some traditional Asian countries but weaker than those of the Western countries. Cross-cultural comparisons are potentially useful.

 It is interesting to see that perceived infringement of personal freedom was not directly associated with social distancing behaviors. It thus did not point in the opposite direction against perceived response efficacy in determining levels of social distancing. One plausible reason for the non-significant association between perceived infringement of freedom and social distancing was that, as previously mentioned, Hong Kong Chinese people are strongly influenced by the traditional Chinese culture that emphasizes collectivism. Some aspects of collectivism have been reported to be associated with specific health-related behaviors (eg, risky sexual behaviors) in previous studies.^45,46^ It may partially explain the cross-country variations in the uptake of preventive measures against COVID-19 (such as social distancing) and the degree of perceived freedom infringement regarding social distancing. Some people might practice social distancing even if they feel that the measure has some infringements of their freedom because of collectivism. Some degrees of personal sacrifice for social good is the norm among Chinese people.^47^ Collectivism seems to have been well observed in China during the pandemic; an illustration is the great support shown to the government despite personal inconvenience during the lockdowns.^48^

 Despite the non-significant association with social distancing behaviors, interestingly, perceived infringement of freedom moderated the significant association between perceived response efficacy and social distancing behaviors. The moderation effect elaborates the relationship between perceived response efficacy and social distancing behaviors. The association was significant only among those who perceived infringement of freedom, but not among those who did not. The data suggest that perceived response efficacy might not influence all people. It might only matter in the presence of a value conflict regarding social distancing. It is plausible that those who possessed a negative value (infringement on personal freedom in this case) might need a stronger reason (perceived response efficacy) to initiate social distancing behaviors. It is noteworthy that the relationship between perceived response efficacy and social distancing might actually depend on the presence of a potentially inherent value conflict (a structural factor) regarding social distancing. Future studies may look at whether other structural moderators (eg, financial loss related to social distancing and collectivism) exist for the association between perceived response efficacy (and also other cognitive factors) and social distancing. However, readers are reminded that the moderation analysis was preliminary and exploratory in nature, and needs to be confirmed by future studies.

 The study has some limitations. First, the study has some methodological limitations. (1) We cannot make causal inferences as this was a cross-sectional study. (2) Social desirability bias and recall bias may exist. (3) The observed effect size of the associations between perceived response efficacy/perceived infringement of personal freedom and social distancing was small (*R*^2^ ranged from 10%-13%). The finding implies that some important factors might not have been included in the present study (eg, perceived risk and perceived susceptibility related to COVID-19, and variables related to social distancing, such as self-efficacy, boredom, and social norms). (4) The response rate was not high, although being comparable to other telephone surveys conducted in Hong Kong^49,50^; characteristics between participants and non-participants may differ. (5) The sample’s age distribution and education levels were similar to those of the census,^51^ although there were slight differences. For instance, the 18-35 age group was under-represented (17.7% versus 22.8%), while the >65 age group was slightly over-represented (25.7% versus 21.2%) in the sample. Besides, people with tertiary education were slightly under-represented in our sample when compared to the census data (25.6% versus 33.1%). Slight biases may exist, which should be normal. (6) The sequence of the question items was fixed and there might be an order effect. Second, some limitations refer to variable selection: (1) The study did not investigate the employment status of participants, which may be an important socio-demographical factor of social distancing. (2) It did not investigate other potential moderators, such as self-control, collectivism, and financial loss due to COVID-19. Third, some contextual limitations are also noteworthy: (1) Perceived infringement of personal freedom was measured by a single item about whether the restriction on gathering size of >4 persons in public areas would infringe personal freedom as the other commonly adopted mandatory governmental social distancing behaviors (eg, stay-at-home order, working from home, and closure of shops and restaurants) were not in place in Hong Kong during the study period. (2) Hong Kong is undergoing socio-political movements and turmoil prior to and during the COVID-19 pandemic, the social distancing measures might have been conflated with the heated political sentiments that the measures were intended to limit political freedom (eg, the right to protest). Interpretations need to take such processes into account.

## Conclusion

 We found high levels of various types of social distancing behaviors and relatively high levels of perceived response efficacy. About 1/5 of the participants were concerned about the infringement of personal freedom due to a social distancing measure. It is interesting that perceived response efficacy did not compete (opposite effects) against each other to affect social distancing. Instead, a significant moderation effect was found. In short, social distancing may involve both public health benefits (eg, perceived response efficacy) and conflicting values (eg, perceived infringement of freedom). It is important to elaborate on the roles of the conflicting values in the relationship between cognitive factors and social distancing behaviors. There is a dearth of similar studies that investigated the role of structural factors and social values in determining social distancing, or how such factors would modify the effects of cognitive factors on social distancing. Such studies are important to understand and promote social distancing to control COVID-19 as it is a controversial public health measure. Social distancing is a new and understudied area of public health research that requires applications of interdisciplinary research frameworks. Cross-cultural studies are of particular importance as values related to COVID-19 (eg, freedom) may be tremendously different across countries; these studies may involve other theoretical frameworks (eg, the Social Cognitive Theory and Self-determination Theory). Given individual variations in collectivism, future studies should also investigate its relationship with perceived freedom infringement, and whether collectivism would serve as another potential moderator of the association between perceived response efficacy and social distancing behaviors. It is a limitation that it was not assessed in the present study.

 All in all, it is important to understand social distancing and related perceptions in their socio-political contexts. The pandemic and its related measures, both governmental and personal ones, have been strongly politicized in the United States and other countries,^52-54^ where polarization toward the use of such measures was severe and social distancing and face-mask use have been used as symptoms of political stance. In Hong Kong, various opposing political parties have been criticizing many governmental measures for controlling COVID-19, such as receiving support for large-scale testing from the mainland Chinese government. Some politicians criticized the gathering size restriction due to economic reasons, but political parties were basically not divided in the views regarding the gathering size restriction policy. The political impacts on the compliance with social distancing measures in Hong Kong might be lower than that of the United States. However, due to the ongoing series of socio-political unrest,^55,56^ the public’s trust in the government has dived deeply. It is possible that the lack of trust and politicization might reduce perceived response efficacy and its effect on mandatory social distancing. Future studies about social distancing in the COVID-19 context need to take the political context into account. Politicization of the pandemic and community response are hence a potentially important and new research direction.

## Acknowledgement

 We would like to thank all the participants for their contributions.

## Ethical issues

 The study was approved by the Survey and Behavioral Research Ethics Committee of the Chinese University of Hong Kong (No. SBRE-19-661).

## Competing interests

 Authors declare that they have no competing interests.

## Authors’ contributions

 Conceptualization: JTFL and YQY; Methodology: YQY and JTFL; Investigation: MMCL; Software: YQY; Formal analysis: YQY; Data curation: YQY; Validation: JTFL; Resources: JTFL; Writing-original draft: YQY and JTFL; Writing-review & editing: YQY and JTFL; Supervision: JTFL; Funding acquisition: JTFL.

## Funding

 The study was supported by internal research funding of the Centre for Health Behaviour Research. The funding source has no role in this study.
